# Sacral dysmorphism and safe zone dimensions: racial, ethnic and sex-based anatomical considerations for percutaneous iliosacral screw placement

**DOI:** 10.1007/s00590-025-04624-7

**Published:** 2025-12-23

**Authors:** Hyewon Kim, David Ahn, Alexis Driscoll, Ahmed Khokhar, Jeremy Hreha, Joseph Galloway, Mark Reilly, Mark Adams

**Affiliations:** 1 Department of Orthopaedics, Rutgers New Jersey Medical School, Newark, USA; 2 Department of Orthopaedics, Lehigh Valley Health Network, Allentown, USA

**Keywords:** Ethnicity, Racial groups, Orthopaedics, Traumatology, Iliosacral screw, Sacral dysmorphism

## Abstract

**Background:**

Percutaneous iliosacral screw fixation is a widely used method for stabilizing posterior pelvic ring injuries, but anatomical variability, particularly sacral dysmorphism, can complicate screw placement. While global data suggest sacral dysmorphism prevalence varies by race and ethnicity, U.S.-based studies have largely focused on predominantly White populations. This study evaluated anatomical variation and sacral dysmorphism prevalence across sex, racial, and ethnic groups in a diverse U.S. trauma population.

**Methods:**

A retrospective analysis was conducted at a Level 1 trauma center, including adults who underwent pelvic CT scans from 2018 to 2020. Exclusion criteria included pelvic/sacral pathology or prior surgery. Demographics collected were age, sex, race, and ethnicity. Sacral dysmorphism was assessed using qualitative criteria, and sacral safe zone dimensions were measured at the first three sacral segments. Statistical analyses included chi-square tests, *t*-tests, ANOVA, and unadjusted odds ratios (OR).

**Results:**

Among 144 patients, 28% had sacral dysmorphism. Prevalence was significantly higher in Hispanic (44%) vs. non-Hispanic (21%) patients (OR = 3.86, *p* = 0.001). Men had larger safe zone dimensions than women at the first (*p* = 0.008) and second (*p* = 0.044) sacral segments. Compared to non-Hispanic women, Hispanic women had smaller dimensions at the first (*p* = 0.030) and larger at the third (*p* = 0.032) sacral segments. White males had significantly larger dimensions at the first (*p* = 0.006) and second (*p* < 0.001) sacral segments than males of other races; White females had larger second sacral segment dimensions than females of other races (*p* = 0.008).

**Conclusions:**

Sacral anatomy varies significantly by sex, race, and ethnicity. Recognizing these differences may aid preoperative planning and reduce the risk of malpositioned iliosacral screws.

**Level of evidence III:**

Retrospective Cohort Study.

## Introduction

Percutaneous iliosacral screw fixation is widely recognized as a standard treatment for unstable posterior pelvic ring injuries, providing outcomes comparable to open screw fixation [1, 2]. However, accurate percutaneous screw placement relies heavily on precise intraoperative fluoroscopy and an in-depth understanding of that sacrum’s complex three-dimensional anatomy on that imaging. Despite established surgical techniques, misplaced screws remain a significant complication, occurring in up to 15% of procedures and potentially leading to neurovascular injuries, particularly involving lumbar and sacral nerve roots [1–5].

Sacral dysmorphism is known to present a challenge to accurate screw placement, with reported prevalence as high as 41% [6–9]. Existing literature suggests variability in sacral morphology across different racial and ethnic groups, notably documented in Indian and East Asian populations [9–12]. However, studies investigating sacral dysmorphism prevalence within the United States have primarily focused on populations that are predominantly White. For example, Woods et al. reported a 39% prevalence in a predominantly White population (only 6% Black), limiting the generalizability of their findings across diverse populations [13].

Given the racial and ethnic diversity of the United States population, a more thorough understanding of variations in sacral morphology as well as osseous fixation pathway dimension is clinically relevant for orthopedic trauma surgeons. There are currently five recognized racial categories (White, Black or African American, Native American or Alaska Native, Asian, Native Hawaiian or Other Pacific Islander) and two recognized ethnicities (people of Hispanic/Latino origin and people not of Hispanic/Latino origin) by the United States Preventative Services Task Force (USPSTF) [14]. Therefore, the purpose of this study was to evaluate anatomical differences in pathway sizes and the prevalence of sacral dysmorphism among multiple races and ethnicities and to compare the sacral osseous fixation corridor dimensions at the first three sacral segments among these various demographic groups. The hypothesis is that sacral dysmorphism prevalence and sacral dimensions would differ significantly by sex, race, and ethnicity.

## Methods

A retrospective cohort analysis was performed at a single level 1 trauma center after Institutional Review Board approval (*study number blinded for review*). Inclusion criteria included patient age over 18 years and available CT pelvis without contrast performed during 2018–2020. Exclusion criteria included patients with pelvic pathologies such as fractures, infection, malignancy, or any previous operative intervention of pelvis and/or sacrum. Demographic data such as age, sex, body mass index (BMI) at time of performed CT, race (White, Black, or Other), and ethnicity (Hispanic, including Spanish, Latino, Central, and South American, or non-Hispanic) were collected. Races other than White and Black were grouped together because of the sample sizes of those respective groups in this studied population.

Sacral dysmorphism was identified based on the inability to safely place transsacral transiliac screws in the upper sacral segment on simulated imaging, consistent with characteristics observed by Routt et al. [5]. Quantitative analysis of sacral dysmorphism was completed by the same trauma fellowship-trained orthopedic surgeon using a Picture Archiving and Communication System (PACS) workstation (GE Medical Systems, Waukeshau, WI). Measurements for safe zone quantification of sacral segment were based on previously described techniques by Gardner et al. [6] and Hwang et al. [7]. The first set of measurements performed was the area of the “bottle neck” region or sacral safe zone area. This was measured using a best fit ellipse tool in PACS at the narrowest cross section of first three sacral segments nerve root tunnel on the sagittal planes parallel to the cardinal axis. The second set of measurements were the safe zone widths at the first three sacral segments on coronal and axial cuts. This was determined by the maximal dimensions of the corridor of bone that was parallel to the horizontal in both planes and passed into the contralateral sacral ala. Sacral measurements were made by a fellowship-trained orthopedic trauma surgeon who was blinded to the demographic characteristics (sex, race, ethnicity) of the patient.

Statistical analysis was conducted using SPSS Version 29.0 (SPSS Inc., Chicago, IL). An a priori power analysis indicated that a total of 78 patients would yield a power of 80% for detecting a sex-based difference in S1 axial width [10]. Normality was confirmed with an F-test. The prevalence of sacral dysmorphism was compared between demographic variables. Continuous variables of age and BMI were compared using independent sample *t*-tests and categorical variables of sex, race, and ethnicity were compared chi-square analysis, or Fisher’s exact test for values < 5. Independent samples *t*-tests were also used to compare sacral safe zone areas and widths by sex. Sacral measurements were also compared between ethnicity and race, stratified by sex, using independent sample *t*-tests and ANOVA respectively. Odds ratios (OR) and 95% confidence intervals (CI) were calculated using unadjusted cross-tabulation. Statistical significance was defined as *p* < 0.05.

## Results

### Demographics

A total of 144 patients were included: 69 males (48%) and 75 females (52%). In terms of ethnicity, 36 (25%) identified as Hispanic and 108 (75%) as non-Hispanic; in terms of race, 28 (19%) identified as White, 70 (49%) as Black, and 46 (32%) as other races. Among the entire cohort, 41 patients (28%) had sacral dysmorphism, while 103 (72%) did not. Among those with sacral dysmorphism, by ethnicity, 18 (44%) were Hispanic and 23 (56%) were non-Hispanic; by race, 8 (20%) were White, 15 (37%) were Black, and 18 (44%) were other races (Table 1).

**Table 1:** Demographic breakdown by average baseline characteristics

Baseline characteristic	Overall (*n* = 144)	%	With Sacral dysmorphism (*n* = 103)	%	Without Sacral dysmorphism (*n* = 51)	%	*p*-Value
Mean age (SD)	52.6 (21.6)		56.6 (23.6)		51.1 (20.7)		0.176
Mean BMI (SD)	26.3 (6.8)		26.6 (5.7)		27.6 (7.2)		0.404
*Sex*							0.238
Male	69	47.9%	16	40.0%	53	51.0%	
Female	75	52.1%	24	60.0%	51	49.0%	
*Ethnicity*							**0.001**
Hispanic	36	25.0%	18	45.0%	18	17.3%	
Non-Hispanic	108	75.0%	22	55.0%	86	82.7%	
*Race*							0.188
Black	70	48.6%	15	37.5%	55	52.9%	
White	28	19.4%	8	20.0%	20	19.2%	
Other	46	31.9%	17	42.5%	29	27.9%	

Bold values are statistically significant with p values < 0.05

There was no significant difference in age between patients with sacral dysmorphism (56.6 ± 23.6 years) and those without (51.1 ± 20.7 years; *p* = 0.176). Similarly, there was no significant difference in BMI between patients with sacral dysmorphism (26.6 ± 5.7) and those without (27.6 ± 7.2; *p* = 0.404). Chi-square analysis showed a significant association between ethnicity and sacral dysmorphism (χ²(1) = 11.8, *p* = 0.001). Hispanic patients had 3.91 times higher odds of sacral dysmorphism than non‐Hispanic patients (OR = 3.91, 95% CI [1.61–9.41]) based on unadjusted cross-tabulation. No significant association was observed between sex and sacral dysmorphism (*p* = 0.238) or between race and sacral dysmorphism (*p* = 0.188). The average area, coronal width, and axial widths of the first three sacral segments safe zones, stratified by sex (Table 2), ethnicity (Table 3), and race (Table 4) are outlined below.

**Table 2:** Sacral Area, coronal width, and axial width breakdown by sex

Level	Sex	Area (mm^2^) $\:\pm\:$ SD	*p*-Value	Coronal Width (mm) $\:\pm\:$ SD	*p*-Value	Axial Width (mm) $\:\pm\:$ SD	*p*-Value
S1	Female	201.5 $\:\pm\:$ 114.1	**0.008**	12.8 $\:\pm\:$ 6.1	0.148	9.3 $\:\pm\:$ 5.2	0.051
	Male	256.6 $\:\pm\:$ 111.3		14.3 $\:\pm\:$ 6.3		11.0 $\:\pm\:$ 5.1	
S2	Female	109.4 $\:\pm\:$ 51.3	**0.044**	12.6 $\:\pm\:\:$3.0	0.103	10.3 $\:\pm\:$ 2.5	0.182
	Male	150.7 $\:\pm\:$ 167.5		13.4 $\:\pm\:$ 3.0		11.1 $\:\pm\:$ 4.9	
S3	Female	29.4 $\:\pm\:$ 21.6	0.252	6.1 $\:\pm\:$ 2.4	0.975	4.0 $\:\pm\:$ 2.2	0.969
	Male	33.6 $\:\pm\:$ 21.6		6.1 $\:\pm\:$ 2.5		4.0 $\:\pm\:$ 2.3	

Bold values are statistically significant with p values < 0.05

**Table 3:** Sacral Area, coronal width, and axial width breakdown by sex and ethnicity

Level	Sex	Ethnicity	Area (mm^2^)$\:\pm\:$SD	*p*-Value	Coronal Width (mm)$\:\pm\:$SD	*p*-Value	Axial Width (mm)$\:\pm\:$SD	*p*-Value
S1	Female	Non-Hispanic	207.8$\:\pm\:$106.2	0.502	13.6$\:\pm\:$6.1	0.109	10.2$\:\pm\:$4.8	**0.030**
		Hispanic	186.6$\:\pm\:$133.0		11.2$\:\pm\:$5.8		7.4$\:\pm\:$5.6	
	Male	Non-Hispanic	267.7$\:\pm\:$99.4	**0.047**	15.0$\:\pm\:$6.2	0.075	11.8$\:\pm\:$4.8	**0.009**
		Hispanic	184.1$\:\pm\:$159.4		11.4$\:\pm\:$6.3		7.6$\:\pm\:$4.9	
S2	Female	Non-Hispanic	103.6$\:\pm\:$50.9	0.153	12.2$\:\pm\:$3.1	0.111	9.9$\:\pm\:$2.7	0.060
		Hispanic	121.8$\:\pm\:$51.0		13.4$\:\pm\:$2.6		11.1$\:\pm\:$1.8	
	Male	Non-Hispanic	150.5$\:\pm\:$183.2	0.990	13.3$\:\pm\:$3.0	0.593	11.1$\:\pm\:$5.3	0.898
		Hispanic	151.2$\:\pm\:$56.1		13.8$\:\pm\:$3.1		11.3$\:\pm\:$2.6	
S3	Female	Non-Hispanic	26.7$\:\pm\:$19.6	0.104	5.8$\:\pm\:$2.3	0.091	3.6$\:\pm\:$2.2	**0.032**
		Hispanic	35.4$\:\pm\:$24.9		6.8$\:\pm\:$2.7		4.8$\:\pm\:$2.1	
	Male	Non-Hispanic	32.4$\:\pm\:$21.7	0.315	5.8$\:\pm\:$2.4	0.054	3.8$\:\pm\:$2.1	**0.045**
		Hispanic	39.3$\:\pm\:$21.2		7.3$\:\pm\:$2.3		5.2$\:\pm\:$2.7	

Bold values are statistically significant with p values < 0.05

**Table 4:** Sacral Area, coronal width, and axial width breakdown by sex and race

Level	Sex	Race	Area (mm^2^) $\:\pm\:$ SD	*p*-Value	Coronal Width (mm) $\:\pm\:$ SD	*p*-Value	Axial Width (mm) $\:\pm\:$ SD	*p*-Value
S1	Female	Black	217.5 $\:\pm\:$ 92.5	0.206	14.2 $\:\pm\:$ 5.7	0.688	10.3 $\:\pm\:$ 4.7	0.621
		White	180.5 $\:\pm\:$ 134.3		12.7 $\:\pm\:$ 7.0		8.8 $\:\pm\:$ 5.6	
		Other	194.3 $\:\pm\:$ 126.9		11.5 $\:\pm\:$ 5.9		8.5 $\:\pm\:$ 5.6	
	Male	Black	252.5 $\:\pm\:$ 119.8	**0.006**	13.6 $\:\pm\:$ 6.2	0.995	10.9 $\:\pm\:$ 5.2	0.946
		White	290.2 $\:\pm\:$ 44.5		16.7 $\:\pm\:$ 6.4		12.4 $\:\pm\:$ 4.8	
		Other	238.3 $\:\pm\:$ 127.0		14.2 $\:\pm\:$ 6.3		10.0 $\:\pm\:$ 5.0	
S2	Female	Black	97.1 $\:\pm\:$ 48.2	0.466	11.9 $\:\pm\:$ 3.3	0.184	9.7 $\:\pm\:$ 2.4	**0.008**
		White	121.5 $\:\pm\:$ 62.1		13.3 $\:\pm\:$ 3.3		11.1 $\:\pm\:$ 3.6	
		Other	117.2 $\:\pm\:$ 47.8		13.0 $\:\pm\:$ 2.4		10.5 $\:\pm\:$ 1.8	
	Male	Black	126.3 $\:\pm\:$ 60.7	**< 0.001**	13.1 $\:\pm\:$ 2.7	0.332	10.4 $\:\pm\:$ 2.7	**< 0.001**
		White	229.4 $\:\pm\:$ 349.9		14.7 $\:\pm\:$ 3.7		13.6 $\:\pm\:$ 9.4	
		Other	139.6 $\:\pm\:$ 52.8		13.0 $\:\pm\:$ 2.7		10.9 $\:\pm\:$ 2.2	
S3	Female	Black	24.4 $\:\pm\:$ 17.2	0.128	5.9 $\:\pm\:$ 2.5	0.998	3.7 $\:\pm\:$ 2.1	0.666
		White	37.9 $\:\pm\:$ 26.6		5.9 $\:\pm\:$ 2.4		4.0 $\:\pm\:$ 2.6	
		Other	30.9 $\:\pm\:$ 22.7		6.4 $\:\pm\:$ 2.5		4.3 $\:\pm\:$ 2.3	
	Male	Black	33.1 $\:\pm\:$ 23.4	0.642	5.8 $\:\pm\:$ 2.4	0.763	4.0 $\:\pm\:$ 2.3	0.775
		White	36.1 $\:\pm\:$ 19.2		5.8 $\:\pm\:$ 2.7		3.5 $\:\pm\:$ 2.0	
		Other	32.8 $\:\pm\:$ 20.4		7.0 $\:\pm\:$ 2.3		4.4 $\:\pm\:$ 2.5	

Bold values are statistically significant with p values < 0.05

## Comparison of sacral safe zone dimensions between men and women

In the first sacral segment, men had significantly larger sacral area (256.6 vs. 201.5 mm^2^, *p* = 0.008) compared to women but not significantly larger coronal width (*p* = 0.148) or axial width (*p* = 0.051). Similarly, in the second sacral segment, men also had significantly larger area (150.7 vs. 109.4 mm^2^, *p* = 0.044), but not significantly larger coronal width (*p* = 0.103) or axial width (*p* = 0.182). In the third sacral segment, no significant differences were found between men and women regarding the area (*p* = 0.252), coronal width (*p* = 0.975), or axial width (*p* = 0.969) (Table 2).

## Comparison of sacral safe zone dimensions between Hispanic and Non-Hispanic women

In the first sacral segment, Hispanic women had significantly smaller axial width (7.4 vs. 10.2 mm; *p* = 0.030) compared to non-Hispanic women. No differences were observed in the second sacral segment between non-Hispanic and Hispanic women regarding the area (*p* = 0.153), coronal width (*p* = 0.111), or axial width (*p* = 0.060). In the third sacral segment, Hispanic women had larger axial width (4.8 vs. 3.6 mm; *p* = 0.032) compared to non-Hispanic women (Table 3).

## Comparison of sacral safe zone dimensions between hispanic and non-hispanic men

In the first sacral segment, Hispanic men had significantly smaller area (184.1 vs. 267.7 mm^2^; *p* = 0.047) and axial width (7.6 vs. 11.8; *p* = 0.009) compared to non-Hispanic men; no significant differences were observed regarding the coronal width (11.4 vs. 15.0 mm; *p* = 0.075). In the second sacral segment, no significant differences were observed between Hispanic and non-Hispanic men regarding the area (151.2 vs. 150.5 mm^2^; *p* = 0.990), coronal width (13.8 vs. 13.3 mm^2^; *p* = 0.593) or axial width (11.3 vs. 11.1 mm; *p* = 0.898). In the third sacral segment, Hispanic men were found to have larger axial width (5.2 vs. 3.8, *p* = 0.045) than non-Hispanic men but no significant differences were observed between the area (39.3 vs. 32.4 mm^2^; *p* = 0.990) or coronal width (7.3 vs. 5.8 mm^2^; *p* = 0.054) (Table 3).

### Comparison of sacral safe zone dimensions between women of black, white, and other race

In the first sacral segment, no significant differences were observed among Black, White, and other race women regarding the area (217.5 vs. 180.5 vs. 194.3 mm^2^; *p* = 0.206), coronal width (14.2 vs. 12.7 vs. 11.5 mm; *p* = 0.688), or axial width (10.3 vs. 8.8 vs. 8.5 mm; *p* = 0.621). Similarly, no significant differences were observed among these three groups in the second sacral segment regarding the area (97.1 vs. 121.5 vs. 117.2 mm^2^; *p* = 0.466) or coronal width (11.9 vs. 13.3 vs. 13.0 mm, *p* = 0.184), but there was a difference found in axial width (9.7 vs. 11.1 vs. 10.5 mm; *p* = 0.008). No significant differences were observed in the third sacral segment regarding the area (24.4 vs. 37.9 vs. 30.9 mm^2^, *p* = 0.128), coronal width (5.8 vs. 5.8 vs. 7.0 mm; *p* = 0.763), or axial width (4.0 vs. 3.5 vs. 4.5 mm; *p* = 0.775) (Table 4).

## Comparison of sacral safe zone dimensions between men of black, white, and other race

In the first sacral segment, no significant differences were observed among Black, White, and other race men regarding the coronal width (13.6 vs. 16.7 vs. 14.2 mm, *p* = 0.995) or axial width (10.9 vs. 12.4 vs. 10.0 mm; *p* = 0.946), but there were differences found in the sacral area (252.5 vs. 290.2 vs. 238.3 mm^2^; *p* = 0.006). In the second sacral segment, there were also differences observed among these three groups regarding the area (126.3 vs. 229.4 vs. 139.6 mm^2^; *p* < 0.001) and axial width (10.4 vs. 13.6 vs. 10.9 mm; *p* < 0.001), but there were no differences in coronal width (13.1 vs. 14.7 vs. 13.0 mm; *p* = 0.332). No significant differences were observed in the third sacral segment regarding the area (33.1 vs. 36.1 vs. 32.8 mm^2^; *p* = 0.632), coronal width (5.8 vs. 5.8 vs. 7.0 mm; *p* = 0.763), or axial width (4.0 vs. 3.5 vs. 4.4 mm; *p* = 0.775) (Table 4).

## Discussion

This study evaluated how sex, race, and ethnicity influence the prevalence of sacral dysmorphism and safe zone dimensions within the first three sacral segments. Several key distinctions were made: (1) Hispanic patients had a significantly higher prevalence of sacral dysmorphism than non-Hispanic patients; (2) sacral safe zone dimensions varied significantly between Hispanic and non-Hispanic patients, particularly in the first and second sacral segments; (3) men had generally larger sacral safe zone dimensions than women, particularly in the first and second sacral segments; (4) White males had larger S1 area, S2 area, and S2 axial widths than males of other races, and White females had larger S2 axial widths than females of other races; and (5) dimensions of the third sacral segment were uniformly small across groups, limiting its utility for iliosacral fixation. The frequency of iatrogenic inaccurate transsacral transiliac screw placement is as high as 59% with permanent nerve injury occurrence reported up to 5%. Increased awareness of these difference may help surgeons anticipate challenges during screw placement.

In dysmorphic sacra, iatrogenic sacral nerve damage from improper screw placement is higher compared to non-dysmorphic sacra [4, 6, 15–17]. This study’s results indicate certain patient populations have higher incidence of dysmorphic sacra, therefore surgeons can appropriately plan for difficult iliosacral screw fixation by anticipating possible need for 3D reconstruction CT scans [3, 18, 19], the use of intraoperative CT, and/or the use of navigation instead of just fluoroscopy [20–23].

The finding that Hispanic patients had significantly greater odds of sacral dysmorphism aligns with existing literature suggesting variability in sacral morphology among different racial and ethnic groups. For instance, Kaiser et al. found an elevated prevalence of sacral dysmorphism (41%) in a diverse urban U.S. trauma population, attributing their higher rates than previously reported (~ 30%) to the ethnic diversity (Asian, African American, Latino) of their patient cohort compared to prior studies predominantly focused on White populations [15]. However, Kaiser et al. acknowledged limitations due to small sample sizes within these ethnic groups. Conversely, Woods et al. reported no significant differences in sacral dysmorphism prevalence across ethnic groups [13]; however, their patient demographics (predominantly White (66%), with fewer Hispanic (26%) and Black (6%) patients) differed notably from ours (approximately 40% Hispanic and 50% Black). These contrasting findings underscore the importance of recognizing population-specific variability in sacral morphology and highlight the necessity for future studies to standardize definitions of race and ethnicity to allow accurate cross-study comparisons.

This investigation identified significant ethnic differences in sacral safe zone dimensions within both sexes. Hispanic women demonstrated smaller first sacral segment dimensions (axial widths), yet larger third sacral segment dimensions compared to non-Hispanic women. Hispanic men similarly had smaller first segment areas and axial widths compared to non-Hispanic men. These ethnic-specific variations suggest distinct anatomical patterns in the Hispanic population that may influence surgical planning for iliosacral screw placement. Notably, prior research from East Asia similarly identified regionally unique sacral morphology and narrower safe zones among Japanese, Taiwanese, and Korean populations compared to Western populations [9, 11, 12, 24]. Trikha et al. [10] observed smaller sacral safe zones among Indian patients compared to Western counterparts, reinforcing the concept that ethnicity significantly influences sacral anatomical variability.

Significant sex-based differences in sacral dimensions were observed: men consistently had larger sacral safe zone areas and widths than women, particularly within the first two sacral segments. These findings are consistent with prior studies demonstrating smaller safe zone dimensions in female patients. For example, Trikha et al. [10] found significantly greater vertical height and anteroposterior width of the first sacral vestibule (S1) in males compared to females, which aligns closely with our findings of sex-specific differences in sacral dimensions. Their study further emphasized the clinical importance of individualized preoperative planning, particularly considering the notably smaller safe zone dimensions observed among women.

Interestingly, our results showed that White males and females had larger S1 and S2 measurements, specifically S1 area, S2 area, and S2 axial width for males and S2 axial width for females, compared to their counterparts of other races. There have been previous studies demonstrating that White females have larger sacral measurements than females of other races [25–27]. However, the existing literature in this specific area remains sparse and varied, partly due to inconsistencies in defining racial groups across studies. For example, Woods et al. categorized “White” and “Black” as ethnicities rather than races [13], whereas this study clearly separated race from ethnicity. As of 2021, USPSTF clearly defined race and ethnicity to acknowledge systemic racism and to address racism and health equity in clinical research [14]. The variability in existing literature highlights a limitation in current orthopedic research; future studies should aim to adhere to these standardized definitions and reporting of racial and ethnic demographics, particularly when investigating anatomical and clinical implications.

This data regarding the third sacral segment (S3) demonstrated consistently smaller safe zone dimensions across all demographic groups, limiting its utility for routine iliosacral screw placement. However, these findings echo recent literature suggesting S3 may still provide supplemental fixation opportunities in select scenarios, particularly in sacral dysmorphism. Specifically, Hwang et al. evaluated the dimensions of the S3 osseous fixation pathway, identifying that although generally smaller than S1 or S2, dysmorphic sacra had significantly larger S3 corridors compared to normal sacra [7]. They reported that approximately 43% of dysmorphic patients could accommodate an S3 screw using a 9-mm threshold, compared to just 10% in normal sacra, emphasizing its utility as an additional fixation site when upper sacral segments are unsuitable or inadequate [7]. Similarly, Eastman et al. demonstrated that a viable S3 fixation pathway for a transiliac-transsacral screw (≥ 7 mm) existed in roughly 15% of all patients, predominantly among those with dysmorphic sacra [28]. However, they underscored caution due to narrower pathways and smaller margins for technical error during screw placement. Collectively, these studies and the findings in this study reinforce that although routine use of the S3 segment for screw fixation is limited by small corridor dimensions, it remains a potentially valuable fixation option in dysmorphic sacra, particularly when upper segments provide insufficient fixation opportunities.

This study has several limitations. Firstly, measurements were conducted by a single orthopedic trauma surgeon. While multiple observers might have increased reliability, the measurement techniques used in this study closely mirrored validated methods used in prior research, supporting the reproducibility of our findings. Additionally, the classification system for ethnicity used (binary: Hispanic or non-Hispanic) oversimplifies a spectrum that includes diverse groups potentially differing in anatomy. Hispanic patients originating from Mexico, for example, may have different morphological characteristics than those from more southern Latin American countries. Future research should pursue increased granularity in ethnic classification, potentially revealing subtle but clinically important variations. Despite these limitations, this study has multiple strengths including a relatively diverse cohort, robust statistical analyses, and clearly defined anatomical measurement methods enhance the reliability and clinical applicability of these findings. Finally, this study was conducted at a single Level 1 trauma center with a finite sample size, which may limit the statistical power of certain subgroup analyses, particularly when stratified simultaneously by sex and race or ethnicity. While our a priori power analysis was based on previously published sex-based differences in sacral morphology, the observed associations, especially the higher prevalence of sacral dysmorphism among Hispanic patients, remained statistically significant despite these limitations, suggesting that these relationships are likely robust. Expanding the cohort to additional centers or years could improve subgroup representation, but the current dataset provides meaningful and novel insight into demographic variation in sacral anatomy. Future multicenter investigations are warranted to validate these findings across larger and more diverse populations.

Importantly, while this study demonstrates significant anatomical differences associated with sex, race, and ethnicity, these demographic variables should not be used in isolation to guide clinical care. Race and ethnicity are sociopolitical constructs that do not reliably predict individual anatomy. Most modern healthcare systems, including those in many democratic nations, do not incorporate race in clinical decision-making, as doing so may perpetuate bias. However, awareness of population-level anatomical variability can enhance surgical education, support implant design, and inform preoperative vigilance—particularly in dysmorphic or anatomically complex cases. These findings are intended to contribute to broader anatomic understanding, not to suggest that demographic labels replace individualized imaging or patient-specific evaluation.

These findings strongly suggest that ethnicity, particularly Hispanic background, is associated with a higher prevalence of sacral dysmorphism and distinct safe zone dimension variations. Sex-based differences were also prominent, whereas White race alone showed some associations with larger sacral measurements. These anatomical differences underscore the clinical necessity of incorporating patient demographics into preoperative planning for percutaneous iliosacral screw fixation, thereby optimizing patient outcomes and minimizing neurovascular complications. Future research should aim to standardize racial and ethnic classifications, facilitating clearer cross-study comparisons and enhancing understanding of how demographic variables influence orthopedic anatomy and clinical outcomes.

## Data Availability

No datasets were generated or analysed during the current study.
